# Optimizing the Shunting Schedule of Electric Multiple Units Depot Using an Enhanced Particle Swarm Optimization Algorithm

**DOI:** 10.1155/2016/5804626

**Published:** 2016-06-29

**Authors:** Jiaxi Wang, Boliang Lin, Junchen Jin

**Affiliations:** ^1^School of Traffic and Transportation, Beijing Jiaotong University, Beijing 100044, China; ^2^Department of Transportation Science, KTH Royal Institute of Technology, Teknikringen 10, 10044 Stockholm, Sweden

## Abstract

The shunting schedule of electric multiple units depot (SSED) is one of the essential plans for high-speed train maintenance activities. This paper presents a 0-1 programming model to address the problem of determining an optimal SSED through automatic computing. The objective of the model is to minimize the number of shunting movements and the constraints include track occupation conflicts, shunting routes conflicts, time durations of maintenance processes, and shunting running time. An enhanced particle swarm optimization (EPSO) algorithm is proposed to solve the optimization problem. Finally, an empirical study from Shanghai South EMU Depot is carried out to illustrate the model and EPSO algorithm. The optimization results indicate that the proposed method is valid for the SSED problem and that the EPSO algorithm outperforms the traditional PSO algorithm on the aspect of optimality.

## 1. Introduction

Artificial intelligence algorithms (e.g., genetic algorithm, simulated annealing algorithm, and particle swarm optimization algorithm) have been an effective and efficient method used for solving large-scale combinational optimization problems arising in the fields of information technology, economic management and transportation, and so forth. Although these algorithms cannot always guarantee the optimal solution, their fast computational speeds have attracted many researchers and engineers. In the area of transportation, the solution space of real-life optimization problems (e.g., network design, train routing, and train timetabling) is extremely huge, even for small-sized instances. As a result, the traditional operational research (OR) methodology is unable to address these problems. In this context, artificial intelligence algorithms are regarded as a practical tool to attack the computational burden. In this study, we consider a typical complex combinational optimization problem of determining an optimal depot shunting schedule and employ one of the most widely used artificial intelligence algorithms, the particle swarm optimization algorithm, to solve this problem.

The shunting schedule of electric multiple units (EMUs) depot determines the time periods of staying on tracks and being shunted between tracks for EMUs. It guides the depot operators to execute maintenance works in a well-ordered way. Therefore, it plays a major role in ensuring safe operations of high-speed trains. Currently, the shunting schedule of EMU depot (SSED) is mainly manually designed by depot dispatchers in the daily operation. Since many influential factors need to be taken into consideration, such as train schedules, track layout of depots, and train routes conflicts, it is a time-consuming and laborious work to develop a well-performed SSED. In practice, the schedule making process always requires several hours of painstaking effort by a team of highly experienced dispatchers. Along with the constantly increasing number of EMUs, the maintenance tasks in depots and the dispatching workloads of the depot staff are getting heavier and heavier. Consequently, it is of high importance to determine an optimal SSED through automatic computing. To tackle this tricky problem, we present a 0-1 programming model and an enhanced particle swarm optimization algorithm. The computational results validate the proposed method and indicate that the enhanced algorithm outperforms the traditional particle swarm optimization algorithm on the aspect of solution quality.

The remainder of the paper is organized as follows. A literature review is presented in Section 2.  Section 3 is the problem description of the SSED. In Section 4, we provide the model formulation and the details of the SSED problem. An enhanced particle swarm optimization algorithm is described to solve the SSED model in Section 5.  Section 6 illustrates the empirical study to test our model and algorithm. Finally, the conclusions and research prospects are given in Section 7.

## 2. Literature Review

Several literatures which are closely related to the SSED problem should be noted. Jacobsen and Pisinger [1] considered the problem of planning the shunting of train units at a railway workshop area. The problem is to schedule the trains to workshops and depot tracks in order to complete the repairs as soon as possible, while avoiding train blockings at the tracks. They presented three heuristic approaches and carried out several computational experiments for realistic instances. Wang et al. [2] established the integer programming models with the depots' marshalling yard arrangements, occupation compatibility of resources, EMU operation, and routine maintenance plans as the main constraints and with minimization of the unnecessary occupation time of the critical track area and costs of shunting paths as the objective functions. They transformed the original problem into a job shop scheduling problem with additional space and time constraints and constructed a topological graph of shunting tasks. They also designed a solution algorithm based on the max-min ant system for the model. Guo et al. [3] proposed an optimization model for the shunting operation plan of EMU running shed. The objective of the model is to minimize the total delay time of EMU in running shed, and the constraints include the number of operation tracks and EMUs, operation sequence, and the occupation time of operation tracks. A microevolution algorithm was developed to solve the problem.

For the train shunting problem, most previous studies have focused on railway station shunting scheduling related problems. In existing literature, the station shunting scheduling problem was also known as the train routing problem (TRP), the train platforming problem (TPP), or the track allocation problem (TAP). Zwaneveld et al. [4, 5] stated that the TRP was a subproblem of the generation of a timetable for a railway company in the STATIONS module of project DONS. They presented a mathematical model formulation based on the node-packing problem (NPP) and then described a solution procedure for the problem, based on a branch-and-cut approach. The approach was tested in an empirical study with data from the stations in the Netherlands. They also discussed the complexity issues. They reached the conclusions that when each train had three routing possibilities, the TRP was NP-complete. However, if each train had at most two routing possibilities, then the TRP could be solved in polynomial time [6]. De Luca Cardillo and Mione [7] formulated the TPP as a general graph-coloring problem that they defined as the* kL*-list *λ* coloring problem. Billionnet [8] used integer programming to reformulate the TPP and solved it with the software XA. Carey and Carville [9] presented the notion that the TPP did not have a clear objective function because it involved multiple train operators with conflicting interests. Therefore, they developed scheduling heuristics analogous to those successfully adopted by train planners using “manual” methods. They tested the model and algorithms by applying them to a typical large station and the results compared well with those found by traditional methods that had not taken cost and preference trade-offs into account. In subsequent studies, they extended this work to a rail corridor [10]. Cui et al. [11] analyzed the hypostasis characteristics of the problem on how to select route in marshalling yard. They established a mathematical model of route dispatching in marshalling station to minimize the weighed summation of all jobs while satisfying dynamic constraints of the contextual working procedure routes. The genetic algorithms for solving problems were put forward as well. Lusby et al. [12] reformulated the TRP as a set-packing model with a resource-based constraint system, which was tighter than the conventional node-packing model, and developed a branch-and-price algorithm that exploited the structure of the set-packing model. Caprara et al. [13] studied a general formulation of the TPP, which contained as special cases all the versions previously considered in the literature as well as a case study from the Italian Infrastructure manager that they addressed. They considered a general quadratic objective function and proposed a new way to linearize it by using a small number of new variables along with a set of constraints that could be separated efficiently by solving an appropriate linear program. Liujiang et al. [14] proposed a bottleneck optimization model to enhance the carrying capacity by reasonably arranging routes and groups of turnouts with the consideration of proportionality and minimized total occupation time. Wu et al. [15] defined the TRP as a track allocation problem. They presented a mean-variance optimization model for solving the TAP that minimized the occupation time costs in groups of turnouts at station bottlenecks. Then, the simulated annealing algorithm was provided to solve this programming. Sels et al. [16] focused on automatically platforming as many trains as possible from two train sets: an already operational set (current set) and a future set, based on the expected traffic increase. Their platforming solution was a mixed integer linear programming model that extended the model of [8] with consideration of route duration differences, as well as with the fictive route concept.

Apart from the conflict graph methodology (which includes the node-packing approach and the graph-coloring approach) and constraint programming approaches, scheduling model and algorithm were also used by researchers to solve the shunting schedule problem [17–19].

Though in-depth studies have been done and great research achievements have been made by researchers, the achievements cannot be applied to this paper directly. There are several research gaps between the present work and previous studies; for example, (i) train routes consist of receiving route and departure route in the TRP while the SSED problem also involves shunting route, which makes the constraints more complex; (ii) in the TRP, the train schedule is given as one of the input conditions, which means the starting and ending times of each route are determinate as long as they obey the schedule; but, in the SSED problem, the starting and ending times of shunting routes are contents of the shunting schedule itself, which greatly enlarges the solution space. It is hard to image how large the solution space will be when the number of EMUs increases and the maintenance procedure gets more complex. As to this point, we can refer to Section 4 for detail. In this paper, we take the uniqueness of the SSED into account and formulate the problem as a 0-1 programming model. Then, an enhanced particle swarm optimization algorithm combining roulette wheel selection operator is proposed to solve this problem. The computational results prove that the proposed model and algorithm are valid for the SSED problem and the enhanced particle swarm optimization algorithm compares well with the traditional particle swarm optimization algorithm on the aspect of optimality.

## 3. Problem Description of the SSED

EMU depots, including operating depots and overhaul depots, are dedicated to the maintenance works of EMUs. According to the maintenance regulations of China high-speed railway, EMU maintenance is divided into five levels based on maintenance cycles and maintenance items. Among them, the first-level maintenance and the second-level maintenance are called operating maintenance while the rest are overhaul maintenance. The first-level maintenance and second-level maintenance are executed in operating depots and the third-level maintenance, fourth-level maintenance, and fifth-level maintenance are carried out in overhaul depots. In this paper, the main focus is on the shunting schedule of EMU first-level maintenance.

**Figure 1:**
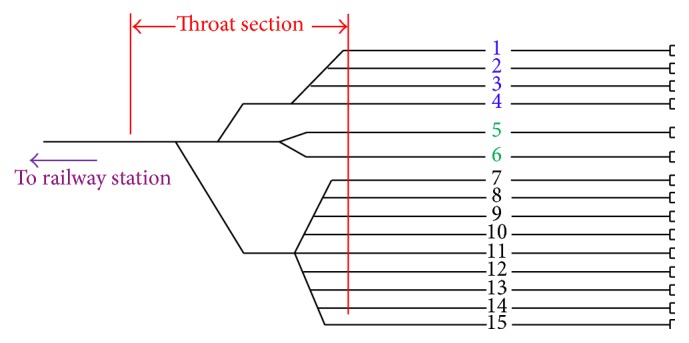
A typical track layout of EMU depot.

Both time and space factors are taken into account when developing maintenance strategies and maintenance regulations. As a result, there are a time cycle and a distance cycle for each level of maintenance. For the first-level maintenance, the time cycle and the distance cycle are two days and 4,000 km, respectively. In other words, when an EMU train's service time from the last maintenance has reached two days or its accumulated mileage from the last maintenance has reached 4,000 km, the train should go to the depot to conduct the first-level maintenance. The procedure of first-level maintenance mainly consists of the inspection process and the washing process. The order of these two processes is not fixed; that is, the inspection process can be done either before or after the washing process. Different processes should be accomplished on the corresponding tracks with specific functions. Specifically, the inspection process should be accomplished on the inspection tracks that are equipped with fault inspection and correction equipment while the washing process should be accomplished on the washing tracks that are equipped with train cleaning equipment (e.g., automatic train washer). Besides, storage tracks are required in order to store trains and serve as the buffer between shunting operations.  Figure 1 shows a typical track layout of EMU depot. In Figure 1, track 1~track 4 (in blue), track 5 and track 6 (in green), and track 7~track 15 (in black) are the inspection tracks, washing tracks, and storage tracks, respectively. The red lines mark the throat section. The tracks and the throat section are all depot resources and may become the bottleneck that determines the depot capacity.

The procedure of the first-level maintenance is described in detail as follows. First, the EMU trains will arrive at the depot at scheduled times. Next, the trains can either be received to inspection tracks to start the inspection process or be assigned to washing tracks to start the washing process. It is also possible that all the inspection tracks and washing tracks are occupied at the moment when an EMU train arrives. In this case, the train has to be received to a storage track to wait for inspecting and washing processes. Then, if a train has completed the inspection process, it will be transferred to a washing track by shunting operation to start the washing process. Similarly, a train will be transferred to an inspection track after finishing the washing process. At last, when a train has completed the two processes, it has to wait for leaving the depot till the scheduled departure time. Regarding waiting for leaving, the train can either be shunted to a storage track or continue to stay on the washing or inspection track depending on whether the tracks are requested by other trains. We will illustrate the above procedure through the following example. Train T1 arrives at the depot at 20:00, and then it is received to track 1 through the throat section (see Figure 1). After finishing a certain length of time (e.g., 120 minutes) of the inspection process, the train will be transferred to track 5 by shunting operation. During the shunting process, the throat section is occupied by the train once again. On track 5, the train will complete the washing process and then will be again shunted to track 7 in order to wait for the departure. That is, assuming that the scheduled departure time is 07:00 the next day, the train will stay on track 7 until 07:00 and leave the depot through the throat section. If the train does not need to wait on the storage track, there are two shunting movements and the throat section is occupied four times considering this typical example. We can see from the whole maintenance procedure that the SSED is actually a program capable of determining when the EMUs should occupy the tracks and when the EMUs should be transferred between tracks. The train routes, guaranteeing all EMUs completing maintenance processes and avoiding conflicts, are determined by the SSED as well.

## 4. Model Formulation

In this section, we will formulate the SSED problem as a 0-1 programming model. This model focuses on the parallel-arrangement layout depot with single throat section. The model aims at determining (i) on which tracks the inspection and washing processes for each EMU are to be completed, (ii) the starting and ending times for each EMU to occupy the tracks, and (iii) during what time period the throat section resource is used by EMUs (i.e., what time to carry out shunting operations). The objective of the model is to minimize the total shunting cost. As shunting costs increase with shunting movements, the objective can be equivalently transformed to minimizing the total number of shunting movements.

### 4.1. Model Assumptions and Notations

First of all, some assumptions are made in our model, including the following:

- The receiving time and dispatching time for each EMU are the same; that is, the occupation times of the throat section are identical when receiving or dispatching trains.
- The shunting movement times between any tracks are the same; that is, the occupation times of the throat section are the same when carrying out shunting operations.
- Delays are not allowed; that is, every EMU arrives at the depot at scheduled time and must be dispatched from the depot at scheduled time.

We then describe the notations used in the model.

First, we have the following sets:

 
*E*: the set of all EMUs needed to complete the first-level maintenance, *E* = {*e*∣1,2, 3,…, *N*_*E*_}, where *N*_*E*_ is the total number of EMUs.
 
*T*_*e*_^*R*^: time period of EMU *e* to be received into the depot. Hence, *T*_*e*_^*R*^ = {*t*∣*t*_*e*_^*A*^ ≤ *t* ≤ *t*_*e*_^*A*^ + *t* ^*R*^}, where *t*_*e*_^*A*^ is the scheduled arrival time of EMU *e* and *t* ^*R*^ is the train receiving running time. During the time period *T*_*e*_^*R*^, the throat section will be occupied.
 
*T*_*e*_^*D*^: time period of EMU *e* to be dispatched out of the depot. Hence, *T*_*e*_^*D*^ = {*t*∣*t*_*e*_^*D*^ − *t* ^*P*^ ≤ *t* ≤ *t*_*e*_^*D*^}, where *t*_*e*_^*D*^ is the scheduled departure time of EMU *e* and *t* ^*P*^ is the train dispatching running time. During the time period *T*_*e*_^*D*^, the throat section will be occupied.
 
*T*_*e*_^*S*^: pure staying time period of EMU *e* at the depot.
 
*T*_*e*_: total time period of EMU *e* staying at the depot. It ranges from the scheduled arrival time to the scheduled departure time, and it consists of three parts: the receiving running time, pure staying time, and the dispatching running time, *T*_*e*_ = *T*_*e*_^*R*^ ∪ *T*_*e*_^*S*^ ∪ *T*_*e*_^*D*^.
 
*T*: time span of the shunting schedule, *T* = ⋃_*e*∈*E*_ *T*_*e*_.
 
*I*: the set of inspection tracks, *I* = {*i*∣1,2, 3,…, *N*_*I*_}, where *N*_*I*_ is the total number of inspection tracks.
 
*W*: the set of washing tracks, *W* = {*w*∣1,2, 3,…, *N*_*W*_}, where *N*_*W*_ is the total number of washing tracks.
 
*S*: the set of storage tracks, *S* = {*s*∣1,2, 3,…, *N*_*S*_}, where *N*_*S*_ is the total number of storage tracks.

The following parameters are considered:

 
*IT*: constant time duration for the inspection process.
 
*WT*: constant time duration for the washing process.
 
*ST*: constant running time for a shunting movement.

Decision variables are as follows:

(1) xeit=1if inspection track i is occupied by EMU e at time t0elseyewt=1if washing track w is occupied by EMU e at time t0elsezest=1if storage track s is occupied by EMU e at time t0elseset=1if the throat section is occupied by EMU e at time t0else.

We define *IT*_*e*_^Start^ as the starting time of EMU *e* staying on the inspection track, *IT*_*e*_^Start^ = min_*t*∈*T*_*e*_^*S*^,*i*∈*I*_⁡{*t*∣*x*_*ei*_^*t*^ = 1}; *IT*_*e*_^End^ as the ending time of EMU *e* staying on the inspection track, *IT*_*e*_^End^ = max_*t*∈*T*_*e*_^*S*^,*i*∈*I*_⁡{*t*∣*x*_*ei*_^*t*^ = 1}; *IT*_*e*_ as total staying time period on inspection tracks of EMU *e*, *IT*_*e*_ = ∑_*t*∈*T*_*e*_^*S*^_∑_*i*∈*I*_ *x*_*ei*_^*t*^; *WT*_*e*_^Start^ as the starting time of EMU *e* staying on washing track, *WT*_*e*_^Start^ = min_*t*∈*T*_*e*_^*S*^,*w*∈*W*_⁡{*t*∣*y*_*ew*_^*t*^ = 1}; *WT*_*e*_^End^ as the ending time of EMU *e* staying on washing track, *WT*_*e*_^End^ = max_*t*∈*T*_*e*_^*S*^,*w*∈*W*_⁡{*t*∣*y*_*ew*_^*t*^ = 1}; and *WT*_*e*_ as the total staying time period on washing tracks of EMU *e*, *WT*_*e*_ = ∑_*t*∈*T*_*e*_^*S*^_∑_*w*∈*W*_ *y*_*ew*_^*t*^. Let *t*_1_, *t*_2_,…, *t*_*n*_ ∈ *T*_*e*_^*S*^ be the time sequence (in an ascending order) of the starting and ending times of shunting movements, {*t*_1_, *t*_2_,…, *t*_*n*_} = {*t*∣|*x*_*ei*_^*t*+1^ − *x*_*ei*_^*t*^| + |*y*_*ew*_^*t*+1^ − *y*_*ew*_^*t*^| + |*z*_*es*_^*t*+1^ − *z*_*es*_^*t*^| = 1}, where *n* equals twice the total number of shunting movements. Among them, *t*_1_, *t*_3_,…, *t*_*n*−1_ are shunting starting times and *t*_2_, *t*_4_,…, *t*_*n*_ are shunting ending times. We define *ST*_*k*_ as the running time of *k*th shunting movement, *ST*_*k*_ = *t*_*k*+1_ − *t*_*k*_, where *k* = 1,2,…, *n*/2.

### 4.2. Formulation

The objective function and constraint conditions can be expressed with a 0-1 programming formulation as follows.

For SSED,

(2) min⁡ Z=∑e∈E ∑t∈TeS∑i∈Ixeit+1−xeit+∑w∈Wyewt+1−yewt+∑s∈Szest+1−zest

(3)  set=1,∀e∈E,  t∈Te−TeS

(4)  ∑i∈Ixeit+∑w∈Wyewt+∑s∈Szest+set=1,∀e∈E,  t∈TeS

(5)  ∑i∈Ixeit+∑w∈Wyewt+∑s∈Szest+set=0,∀e∈E,  t∈T−Te

(6)  ∑e∈Exeit≤1,∀i∈I,  t∈T

(7)  ∑e∈Eyewt≤1,∀w∈W,  t∈T

(8)  ∑e∈Ezest≤1,∀s∈S,  t∈T

(9)  ∑e∈Eset≤1,∀t∈T

(10)  ITe≥IT,∀e∈E

(11)  WTe≥WT,∀e∈E

(12)  ITe=ITeEnd−ITeStart,∀e∈E

(13)  WTe=WTeEnd−WTeStart,∀e∈E

(14)  STk≥ST,∀k∈1,2,…,n2.

As mentioned before, the objective of the SSED problem is to minimize the total number of shunting movements. In fact, a shunting movement corresponds to two state transitions of the depot tracks: (i) from the occupied state to the unoccupied state of one track and (ii) from the unoccupied state to the occupied state of another track. Therefore, the number of shunting movements equals half the number of state transitions of tracks. Certainly, there will be state transitions when trains arrive at the depot or depart from the depot and these state transitions are inevitable and not included in the objective function. The objective function of the SSED model is expressed by (2).

The constraints of this model are defined in formulae (3) to (14). Constraint (3) ensures that the throat section is occupied during the EMU train receiving and dispatching process. Constraint (4) guarantees that the EMUs will occupy a track or the throat section during their pure staying time periods. Constraint (5) ensures that EMUs will occupy neither tracks nor the throat section outside the staying time period. Constraints (6) to (8) are the track conflict constraints; that is, a track can only be occupied by one train at a certain time. Constraint (9) is the throat section conflict constraint; that is, the throat section can only be occupied by one train at a certain time. This constraint is also called the train route conflict constraint since the train routes (including shunting routes, receiving routes, and departure routes) will occupy the throat section when carrying out shunting, receiving, and departure operations. Constraint (10) ensures that the total dwell time on inspection tracks should not be less than the constant time duration for the inspection process while constraint (11) ensures that the total dwell time on washing tracks should not be less than the constant time duration for the washing process. Constraint (12) ensures that inspection process is an uninterrupted process from its starting time to the ending time, and constraint (13) ensures that the washing process is also an uninterrupted process. Constraint (14) ensures that the shunting running time should not be less than the constant running time for a shunting movement.

We can see from the SSED model that the number of all decision variables equals |*T*| · *N*_*E*_ · *N*_*M*_ + |*T*| · *N*_*E*_ · *N*_*W*_ + |*T*| · *N*_*E*_ · *N*_*S*_ + |*T*| · *N*_*E*_ = |*T*| · *N*_*E*_ · (*N*_*M*_ + *N*_*W*_ + *N*_*S*_ + 1), that is, the product of three factors: the time span of the shunting schedule, the number of EMUs, and the number of depot resources (including depot tracks and the throat section). When the orders of magnitude of the three factors are, respectively, 10^3^, 10^1^, and 10^1^, the order of magnitude of decision variables will reach 10^5^. Such a huge search space will bring about great trouble to solve the SSED problem.

## 5. Optimization Using Enhanced Particle Swarm Optimization Algorithm

The SSED problem is NP-hard, which is not easy to be solved by traditional operational research methodology. In practice, researchers usually adopt heuristic algorithms. In this study, we will use an enhanced particle swarm optimization algorithm to solve the SSED problem.

### 5.1. Traditional Particle Swarm Optimization Algorithm

The particle swarm optimization (PSO) algorithm was first proposed by Kennedy and Eberhart in 1995 [20, 21]. It is a population based stochastic optimization technique motivated by social behavior of organisms such as bird flocking and fish schooling. The algorithm was originally used in continuous optimization. In later studies, it expanded to the field of combinatorial optimization. Due to its simple form and effective performance, the PSO algorithm has got great attention of researchers.

The basic update equations of the velocity and position of the particles are as follows:

(15) vidn+1=vidn+c1ξpidn−xidn+c2ηpgd−xidn,

(16) xidn+1=xidn+vidn+1,

where *v*_*id*_^*n*^ is the velocity of *d*th dimension of *i*th particle at *n*th iteration, *x*_*id*_^*n*^ is the position of *d*th dimension of *i*th particle at *n*th iteration, *p*_*id*_^*n*^ is the position of *d*th dimension of the best previous *i*th particle, *p*_*gd*_ is the position of *d*th dimension of the globally best particle, *c*_1_ and *c*_2_ are acceleration coefficients, and *ξ* and *η* are two random numbers in the range [0,1].

In order to improve the convergence performance of the original optimizer, a new parameter called the inertia weight was introduced. This new version of the algorithm was known as the standard particle swarm optimization, in which the velocity update equation was reexpressed by

(17) $$\begin{array}{c} v_{id}^{n+1}=\omega v_{id}^{n}+c_{1}\xi \left(\;p_{id}^{n}-x_{id}^{n}\;\right)+c_{2}\eta \left(\;p_{gd}-x_{id}^{n}\;\right), \end{array}$$

where *ω* is the inertia weight. Many studies show that dynamically adjusting the inertia weight is conducive to balance the exploration (global search) ability and exploitation (local search) ability of the searching process, such as linearly decreasing, fuzzy adaptive, and random inertia weight. In this study, we will adopt the linearly decreasing inertia weight. By linearly decreasing the inertia weight from a relatively large value to a small value through the course of the PSO run, the PSO tends to have more global search ability at the beginning of the run while having more local search ability near the end of the run. We assume that the inertia weight ranges from *ω*_min_ to *ω*_max_ and the maximum iteration is Iter_max_; then the value of inertia weight at *n*th iteration can be expressed by

(18) $$\begin{array}{c} \omega_{n}=\omega_{max}-\frac{\omega_{max}-\omega_{min}}{{Iter}_{max}}n. \end{array}$$

In this way, the velocity update equation can be reformulated as

(19) $$\begin{array}{c} v_{id}^{n+1}=\omega_{n}v_{id}^{n}+c_{1}\xi \left(\;p_{id}^{n}-x_{id}^{n}\;\right)+c_{2}\eta \left(\;p_{gd}-x_{id}^{n}\;\right). \end{array}$$

In this study, we define this version of PSO, in which the velocity and position are, respectively, updated by (19) and (16), as the traditional particle swarm optimization (TPSO) algorithm.

### 5.2. Enhanced Particle Swarm Algorithm

The TPSO algorithm owns the advantage of fast convergence, especially in early stage of the iteration process. However, the algorithm performance is sensitive to its parameters, for example, the inertia weight and the acceleration coefficient. Thus, improper parameters will cause phenomena like divergence and low computational accuracy. Some researchers turned to improve the algorithm performance from the aspect of parameter selection (see the review by García-Gonzalo and Fernández-Martínez [22]). In addition, some other researchers combined other heuristic algorithms to the PSO algorithm and proposed new hybrid algorithms, for example, Firouzi et al. [23], Sun et al. [24], and Li et al. [25]. We have noticed that if a relatively superior particle is gained in the early stage of the PSO run, *p*_*gd*_ will not be updated unless a better particle appears. As a result, other particles will move towards the superior particle in the subsequent run, which is more likely to make the algorithm get trapped into local optima. This motivates us to modify the velocity update equation to improve the exploration ability in early stage of PSO. In this study, we combine the roulette wheel selection operator of the genetic algorithm (GA) with the velocity update equation of the PSO algorithm to improve the global search ability and avoid local optima.

We define Fit(*x*) as the fitness function (the expression of the function will be given below) of the PSO and *G*(*x*) as the fitness transformation function. The relationship between these two functions is as follows:

(20) $$\begin{array}{c} G\left(\;x_{i}^{n}\;\right)=\frac{\sum_{i=1}^{m}Fit\left(x_{i}^{n}\right)}{Fit\left(x_{i}^{n}\right)}, \end{array}$$

where *m* is the population size of the PSO and *x*_*i*_^*n*^ is the position of *i*th particle at *n*th iteration. By normalizing the transformed fitness set {*G*(*x*_1_^*n*^), *G*(*x*_2_^*n*^),…, *G*(*x*_*m*_^*n*^)}, we can obtain the normalized fitness set {*g*(*x*_1_^*n*^), *g*(*x*_2_^*n*^),…, *g*(*x*_*m*_^*n*^)}. Next, we pick a random number *θ* in [0,1] and check whether the expression ∑_*i*=1_^*k*^ *g*(*x*_*i*_^*n*^) ≤ *θ* < ∑_*i*=1_^*k*+1^ *g*(*x*_*i*_^*n*^) is true as index *k* increases from 1. When the expression is true, stop checking and output *x*_*i*_^*n*^. Now we have finished executing the selection operator.

After obtaining the “best” particle selected by the selection operator, we can establish the enhanced particle swarm optimization algorithm by replacing *p*_*gd*_ with *x*_*kd*_^*n*^, where *x*_*kd*_^*n*^ is the *d*th dimension of *x*_*k*_^*n*^. Thus, the velocity update function can be reexpressed by

(21) $$\begin{array}{c} v_{id}^{n+1}=\omega_{n}v_{id}^{n}+c_{1}\xi \left(\;p_{id}^{n}-x_{id}^{n}\;\right)+c_{2}\eta \left(\;x_{kd}^{n}-x_{id}^{n}\;\right). \end{array}$$

We will use (21) to update the particle velocity in the early stage of the PSO, and we will choose (19) near the end of the run. In this paper, we define this new version of PSO as the enhanced particle swarm optimization (EPSO) algorithm.

### 5.3. PSO-Based Solution Express Method

**Figure 2:**
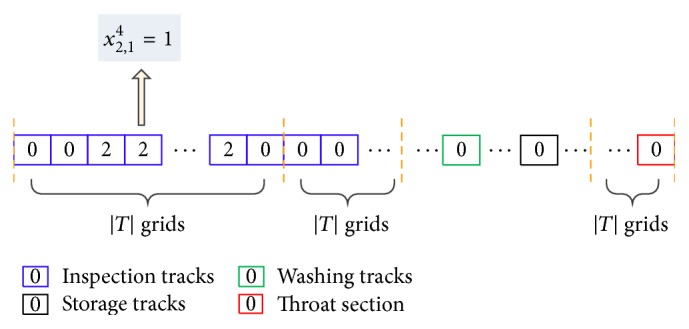
Solution express method.

It has been a crucial step to make the particle of the PSO and the solution of a certain problem correspond with each other. There are three or two indexes of the decision variables in the SSED model, which makes it tricky to express the solutions as particles. In this study, we let each dimension of the particle correspond with one time unit (one minute), the total number of dimensions correspond with the product of shunting schedule time span and the number of depot resources, and the value of each dimension correspond with the EMU number. The detailed description is shown in Figure 2 with the help of schematic diagram.

In Figure 2, each grid refers to a dimension of the particle and the grid chain represents a particle (solution). Thus, the total number of grids equals the total number of dimensions, that is, the product of shunting schedule time span and the number of depot resources. The number inside the grid represents the EMU number. In this way, the location of the grid records both the space information and the time information. For instance, if EMU 2 occupies inspection track 1 at the fourth minute, that is, *x*_2,1_^4^ = 1, the value of the fourth grid will be 2 (see Figure 2). The general case is as follows: the decision variable *x*_*ei*_^*t*^ = 1 corresponds to the (|*T*| × (*i* − 1) + *t*)th grid and its value is *e*.

### 5.4. Fitness Function

In the EPSO algorithm, the fitness function consists of the objective function and the penalty function. It is expressed by

(22) FitX=λ1ZX+λ2∑e∈Emax⁡0,MT−MTe+λ3∑e∈Emax0,WT−WTe,

where **X** = {*x*_*ei*_^*t*^, *y*_*ew*_^*t*^, *z*_*es*_^*t*^, *d*_*e*_^*t*^}  ∀*e* ∈ *E*, *i* ∈ *I*, *w* ∈ *W*, *s* ∈ *S*, *t* ∈ *T*, and  *λ*_1_, *λ*_2_, and *λ*_3_ are positive parameters.

### 5.5. EPSO Structure

Similar to the TPSO algorithm, the structure of the EPSO algorithm is as follows.

**Step 1 (initialization).**

This process is to initialize the velocity and position of all particles. By initializing the particle's position, we can obtain an initial solution. First, we set all decision variables to 0. Then, for all *e* ∈ *E*, select a track randomly from the unoccupied tracks and set the corresponding variable to 1. For example, if EMU 1 is assigned to inspection track 2, the variables *x*_1,2_^*t*^  ∀*t* ∈ *T*_1_ will be set to 1. Next, traverse the EMU set *E* to ensure all EMUs have been assigned to certain track. As for the initial velocity of the particles, we set it as a random number between −*v*_max_ and *v*_max_, where *v*_max_ is the maximum allowable velocity. In our study, we set *v*_max_ to 0.9*N*_*E*_.

**Step 2 (calculation).**

This step is to calculate the fitness of each particle and obtain the value of the best previous particle, globally best particle *p*_*gd*_, and the best particle selected by the selection operator.

**Step 3 (update).**

This step aims at generating neighborhood solutions by updating the velocity and position of the particles according to (16), (19), and (21).

**Step 4 (adjustment).**

In this step, we will adjust the neighborhood solutions to make them feasible. The detail of the adjustment procedure will be presented below.

**Step 5 (stop or not).**

This step is to determine whether the execution of this algorithm should be stopped at the current iteration. If the loop termination condition is not satisfied, the execution should return to Step 2; otherwise, the execution will be stopped. In this paper, the loop termination condition is reaching the maximum iteration Iter_max_.

**Figure 3:**
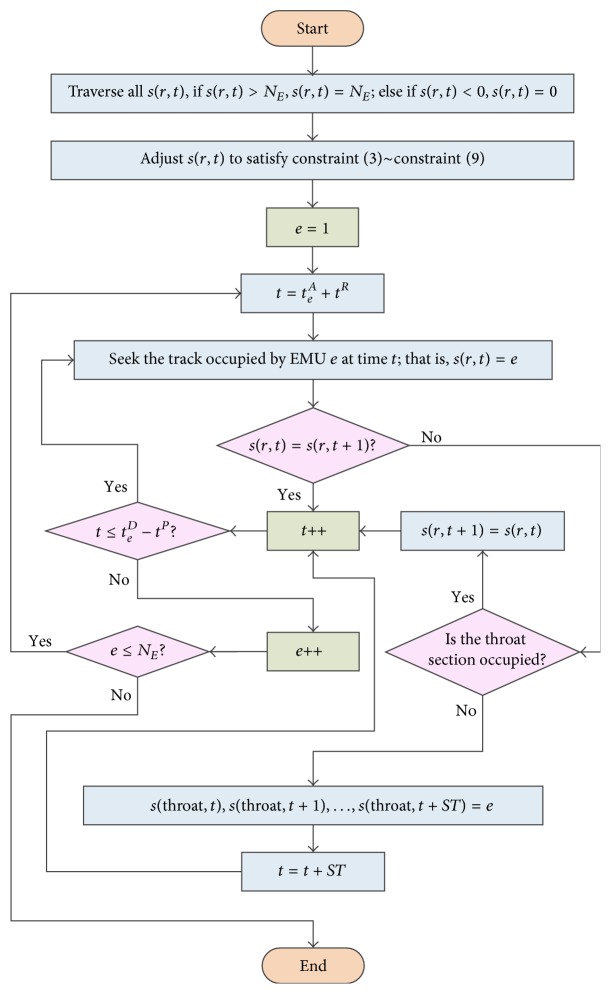
Flow chart of the adjustment procedure.

The neighborhood solutions generated in Step 3 are usually infeasible; for example, the staying time on inspection tracks is less than the constant time duration for the inspection process. As a result, some adjustment operations are necessary to make them feasible, which challenges the algorithm in solving the problem. For illustration purposes, we define *s*(*r*, *t*) as the state of the grids (see Figure 2), where *r* is the track number or the throat section; for example, *s*(*r*, *t*) = *n* means track *r* is occupied by EMU *n* at time *t*. The detailed adjustment procedure of neighborhood solutions is shown in Figure 3.

We can see from Figure 3 that we first adjust the neighborhood solutions to satisfy constraint (3)~constraint (9). In this way, there is no track conflict or throat section conflict. Then, in order to ensure that there must be a shunting movement when *s*(*r*, *t*) ≠ *s*(*r*, *t* + 1), we will check the track's states of every two neighbor minutes. If the two states are not the same, we will check whether the throat section is unoccupied. If it is unoccupied, then a shunting operation can be carried out; otherwise, let *s*(*r*, *t* + 1) be equal to *s*(*r*, *t*). After all EMUs have been traversed, constraint (10)–constraint (14) will be satisfied naturally. Since Figure 3 is the adjustment process of one particle (solution), *m* times of this process need to be repeated if the population size is *m*.

## 6. Case Study

In this section, a case from Shanghai South Depot is carried out to illustrate the application of the proposed model and algorithm.

### 6.1. Case Description and Input Data

Shanghai South Depot, located in Xuhui District, Shanghai, was founded in 2007 and has become one of the first depots built in China high-speed railway system. It mainly undertakes the maintenance tasks of EMUs running on Beijing-Shanghai high-speed line. The depot owns an area of 132,000 m^2^ and its track layout is of parallel-arrangement type with a single throat section (see Figure 1). There are four inspection tracks, two washing tracks, and nine storage tracks in the depot. Every night, an average of eight EMUs return to the depot to complete the first-level maintenance. The time schedule of the EMUs is shown in Table 1.

In order to ensure all EMUs can complete all processes of the first-level maintenance during their staying times, we give the EMUs different priorities based on the sorted staying time. To be more specific, the shorter the time for which EMU stays, the higher the priority it will have. A higher priority means the EMU will be processed earlier in the EPSO run.

The values of the parameters used in our model are listed in Table 2.

And the values of the parameters of the EPSO algorithm are shown in Table 3.

We set the parameters values of the SSED model based on China Railway High-speed (CRH) maintenance regulations and practical experience in Shanghai South Depot while we set the EPSO algorithm's parameters values based on our computational experiments.

### 6.2. Computational Results and Analysis

The proposed EPSO algorithm is coded in Microsoft Visual C++ 6.0 and runs on a computer with an Intel(R) Core(TM) i3-3220 3.30 GHz CPU and 4.00 GB RAM. The program runs for 420 seconds on this computer; the best fitness function value and the corresponding minimum objective function value are 22,400 and 32, respectively. The computational results are shown in Table 4.

As we can see from Table 4, all EMUs have completed the processes of the first-level maintenance. The average staying time of all EMUs on inspection tracks is 174.5 minutes while the average staying time on washing tracks is 94.9 minutes. The total number of shunting movements is 16 and the average number is 2. There are all in all 11 utilized tracks. If we take track utilization amount and balanced occupation objectives into account, the results can be further optimized. We may identify these as our future research work. The throat section is occupied 32 times while the accumulative occupying time and occupancy rate are 112 minutes and 14.7%, respectively.

**Figure 4:**
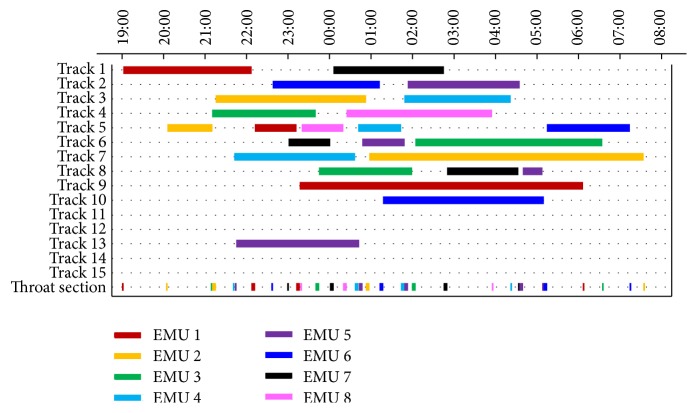
The depot shunting schedule.

To give an insight into the first-level maintenance process of the EMUs, we draw the results in Figure 4 which is called the shunting schedule of EMU depot. In Figure 4, the horizontal axis is the time axis while the vertical axis is the location of the tracks and the throat section. Each EMU is painted with different colors, and the length of colored bars represents the staying time on tracks or occupying time in throat section of the corresponding EMU. We can see from Figure 4 that there are four tracks (tracks 11, 12, 14, and 15) that have never been occupied. These tracks may be used for storing spare EMUs or serve as reserve depot capacity to meet peak maintenance demand.

**Figure 5:**
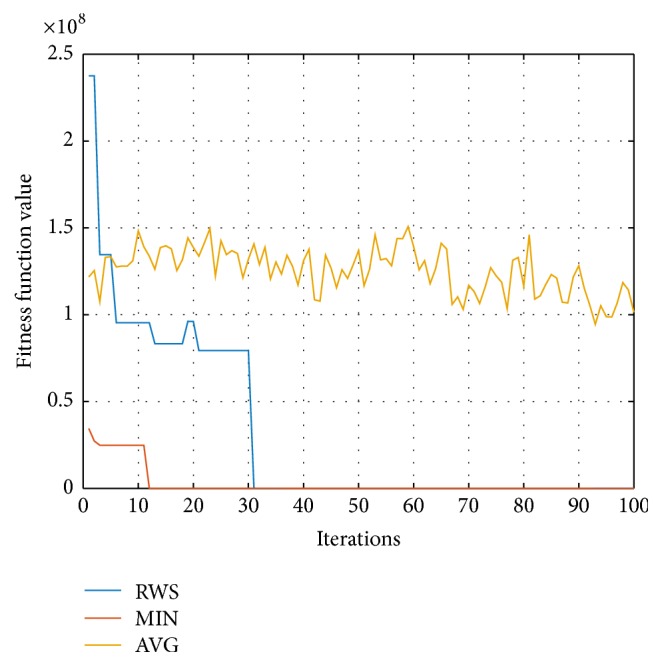
The iteration process.

The iteration process is shown in Figure 5.

In Figure 5, the blue curve represents the “best” particle selected by roulette wheel selection (RWS) operator at each iteration; the red curve is the minimum fitness function value at each iteration; and the yellow curve means the average fitness function value of all particles at each iteration. We can see from the figure that when the EPSO run reaches 31 iterations, the fitness function curves of the RWS and the MIN converge as a single curve. This is because we update the particle velocity based on (21) in the previous 30 iterations while we use (19) as the velocity update equation in the remaining 70 iterations. Since the RWS operator could accept a less-than-optimal solution at a certain probability, the RWS curve may get raised. And this is the reason why the RWS curve has a bulge near the 20th iteration.

### 6.3. Comparison with Traditional Particle Swarm Optimization

In order to test the performance of the EPSO algorithm, comparisons with the TPSO algorithm in several aspects have been made. We focus on the above case and run these two algorithms ten times, respectively. The computational results are shown in Table 5.

We can see from Table 5 that the percent change of the average solution time between EPSO and TPSO is −0.3%, which reflects the notion that they are almost of the same performance on the aspect of computational efficiency. On the aspect of optimality, the average fitness function value of the best solution of the EPSO decreases by 5,600 compared to the TPSO algorithm, that is, a 15.8 percent reduction. In addition, the best solution's fitness function value of the EPSO is better than of the TPSO. These two criterions prove that the EPSO algorithm has an improvement in both optimality and exploration ability. Moreover, the range of the fitness function value of the EPSO algorithm is 38,200 − 22,400 = 15,800 while it is 60,650 − 27,400 = 33,250 for the TPSO algorithm, a 52.5 percent reduction. Meanwhile, the change of fitness function value variance has reached 74.3% between the two algorithms. These two criterions not only indicate the higher robustness but also show the better convergence performance of the EPSO algorithm.

In summary, the EPSO compares well with the TPSO on the aspect of optimality.

## 7. Concluding Remarks

In this study, we first give a formal definition of the SSED problem and present a 0-1 programming model for the problem. The objective of the model is to minimize the total number of shunting movements, and the constraints include the shunting running time, the inspection and washing process time, shunting routes conflicts, track occupation conflicts, and the EMU train schedule. Secondly, we propose an EPSO algorithm by introducing the roulette wheel selection operator. The modification aims at avoiding the defect of falling into local optima easily of the TPSO algorithm. Thirdly, we conduct a real-world instance from Shanghai South Depot of China to illustrate the proposed model and algorithm. The computational results indicate that our method can provide quality solutions with high speed. Finally, we also carry out a comparative study between the TPSO and the EPSO algorithm. The computational results show that the EPSO algorithm outperforms the TPSO algorithm on the aspect of optimality. The proposed method not only can efficiently provide the depot dispatchers with well-performed shunting schedules, but also could be an effective way to utilize the limited depot resources (or maintenance capacity).

However, several improvements could warrant further research. First, the proposed model and algorithm are only suitable for the parallel-arrangement-type layout depot, which has limited its applicability. Therefore, how to reformulate the model and redesign the algorithm to extend them to other types of depot track layout is one of the areas for further research. Furthermore, the solution space will become too huge to deal with as the number of EMUs increases. Hence, how to further improve the computational efficiency of the algorithm to be qualified for large-scale problems is another future research work.

## Figures and Tables

**Table 1:** The time schedule for EMUs.

EMU number	Arrival time	Departure time (the next day)	Total staying time (minutes)
EMU 1	19:00	06:11	671
EMU 2	20:05	07:40	695
EMU 3	21:10	06:40	570
EMU 4	21:42	04:27	405
EMU 5	21:45	05:13	448
EMU 6	22:38	07:20	522
EMU 7	23:01	04:38	337
EMU 8	23:20	04:00	280

**Table 2:** Parameter values of the SSED model.

Parameter	*IT*	*WT*	*ST*	*t* ^*R*^	*t* ^*D*^

Value (minutes)	150	60	5	2	2

**Table 3:** Parameter values of the EPSO algorithm.

Parameter	*m*	*c*_1_	*c*_2_	*ω*_max_	*ω*_min_	Iter_max_

Value	30	2	2	1.85	0.15	100

**Table 4:** Computational results.

EMU number	Staying time on inspection track (minutes)	Staying time on washing track (minutes)	Number of shunting movements
EMU 1	186	60	2
EMU 2	218	65	2
EMU 3	150	271	2
EMU 4	154	62	2
EMU 5	162	61	3
EMU 6	155	120	2
EMU 7	160	60	2
EMU 8	211	60	1
	Avg. = 174.5	Avg. = 94.9	Avg. = 2
			Sum = 16

**Table 5:** Computational results of the EPSO and TPSO.

Criterion	EPSO	TPSO	Change	Percent change
Average solution time (seconds)	418.2	419.4	−1.2	−0.3%
Average fitness function value	29,890	35,490	−5,600	−15.8%
Best fitness function value	22,400	27,400	−5,000	−18.2%
Worst fitness function value	38,200	60,650	−22,450	−37.0%
Fitness function value variance	25,266,400	98,223,400	−72,957,000	−74.3%

## References

[1] Jacobsen P. M., Pisinger D. (2011). Train shunting at a workshop area. *Flexible Services and Manufacturing Journal*.

[2] Wang Z.-K., Shi T.-Y., Zhang W.-J., Wang H. (2013). Model and algorithm for optimized formulation of scheduled shunting operation plans of electric multiple units depots. *Journal of the China Railway Society*.

[3] Guo X., Song R., Li H., Chen S., Liu X. (2016). Optimization of shunting operation plan of electric multiple unit running shed. *China Railway Science*.

[4] Zwaneveld P. J., Kroon L. G., Romeijn H. E. (1996). Routing trains through railway stations: model formulation and algorithms. *Transportation Science*.

[5] Zwaneveld P. J., Kroon L. G., Van Hoesel S. P. M. (2001). Routing trains through a railway station based on a node packing model. *European Journal of Operational Research*.

[6] Kroon L. G., Romeijn H. E., Zwaneveld P. J. (1997). Routing trains through railway stations: complexity issues. *European Journal of Operational Research*.

[7] De Luca Cardillo D., Mione N. (1998). *kL*-list *λ* colouring of graphs. *European Journal of Operational Research*.

[8] Billionnet A. (2003). Using integer programming to solve the train-platforming problem. *Transportation Science*.

[9] Carey M., Carville S. (2003). Scheduling and platforming trains at busy complex stations. *Transportation Research Part A: Policy and Practice*.

[10] Carey M., Crawford I. (2007). Scheduling trains on a network of busy complex stations. *Transportation Research Part B: Methodological*.

[11] Cui B., Ma J., Zhang P. (2007). An optimization algorithm for route dispatching in marshalling station. *China Railway Science*.

[12] Lusby R., Larsen J., Ryan D., Ehrgott M. (2011). Routing trains through railway junctions: a new set-packing approach. *Transportation Science*.

[13] Caprara A., Galli L., Toth P. (2011). Solution of the train platforming problem. *Transportation Science*.

[14] Liujiang K., Jianjun W., Huijun S. (2012). Using simulated annealing in a bottleneck optimization model at railway stations. *Journal of Transportation Engineering*.

[15] Wu J. J., Kang L. J., Sun H. J., Jia X. L. (2013). Track allocation optimization in railway station: mean-variance model and case study. *Journal of Transportation Engineering-ASCE*.

[16] Sels P., Vansteenwegen P., Dewilde T., Cattrysse D., Waquet B., Joubert A. (2014). The train platforming problem: the infrastructure management company perspective. *Transportation Research Part B: Methodological*.

[17] Li W., Wang W., Cheng S. (2000). Scheduling model and algorithm of using up-and-down lines on railway marshalling station. *Systems Engineering-Theory & Practice*.

[18] Zhang Y., Lei D., Liu M. (2010). Scheduling model and algorithm for track application in railway station. *China Railway Science*.

[19] Zhang Y.-G., Lei D.-Y., Tang B., Wang X.-Y. (2011). Due windows scheduling model and algorithm of track utilization in railway passenger stations. *Journal of the China Railway Society*.

[20] Eberhart R., Kennedy J. A new optimizer using particle swarm theory.

[21] Kennedy J., Eberhart R. Particle swarm optimization.

[22] García-Gonzalo E., Fernández-Martínez J. L. (2012). A brief historical review of particle swarm optimization (PSO). *Journal of Bioinformatics and Intelligent Control*.

[23] Firouzi B. B., Sadeghi M. S., Niknam T. (2010). A new hybrid algorithm based on PSO, SA, and K-means for cluster analysis. *International Journal of Innovative Computing, Information and Control*.

[24] Sun Y., Lang M., Wang D., Liu L. (2014). A PSO-GRNN model for railway freight volume prediction: empirical study from China. *Journal of Industrial Engineering and Management*.

[25] Li X., Xu J., Yang Y. (2015). A chaotic particle swarm optimization-based heuristic for market-oriented task-level scheduling in cloud workflow systems. *Computational Intelligence and Neuroscience*.

